# Practice development plans to improve the primary care management of acute asthma: randomised controlled trial

**DOI:** 10.1186/1471-2296-8-23

**Published:** 2007-04-24

**Authors:** Juliet M Foster, Gaylor Hoskins, Barbara Smith, Amanda J Lee, David Price, Hilary Pinnock

**Affiliations:** 1 Dept of General Practice and Primary Care, University of Aberdeen, Foresterhill Health Centre, Westburn Road, Aberdeen, AB25 2AY, UK; 2 Tayside Centre for General Practice, Community Health Sciences, University of Dundee, Kirsty Semple Way, Dundee, DD2 4BF, UK; 3 Department of Community Health Sciences: GP Section, University of Edinburgh, 20 West Richmond St, Edinburgh, EH8 9DX, UK

## Abstract

**Background:**

Our professional development plan aimed to improve the primary care management of acute asthma, which is known to be suboptimal.

**Methods:**

We invited 59 general practices in Grampian, Scotland to participate. Consenting practices were randomised to early and delayed intervention groups. Practices undertook audits of their management of all acute attacks (excluding children under 5 years) occurring in the 3 months preceding baseline, 6-months and 12-months study time-points. The educational programme [including feedback of audit results, attendance at a multidisciplinary interactive workshop, and formulation of development plan by practice teams] was delivered to the early group at baseline and to the delayed group at 6 months. Primary outcome measure was recording of peak flow compared to best/predicted at 6 months. Analyses are presented both with, and without adjustment for clustering.

**Results:**

23 consenting practices were randomised: 11 to early intervention. Baseline practice demography was similar. Six early intervention practices withdraw before completing the baseline audit. There was no significant improvement in our primary outcome measure (the proportion with peak flow compared to best/predicted) at either the 6 or 12 month time points after adjustment for baseline and practice effects. However, the between group difference in the adjusted combined assessment score, whilst non-significant at 6 months (Early: 2.48 (SE 0.43) vs. Delayed 2.26 (SE 0.33) p = 0.69) reached significance at 12 m (Early:3.60 (SE 0.35) vs. Delayed 2.30 (SE 0.28) p = 0.02).

**Conclusion:**

We demonstrated no significant benefit at the a priori 6-month assessment point, though improvement in the objective assessment of attacks was shown after 12 months. Our practice development programme, incorporating audit, feedback and a workshop, successfully engaged the healthcare team of participating practices, though future randomised trials of educational interventions need to recognise that effecting change in primary care practices takes time. Monitoring of the assessment of acute attacks proved to be a feasible and responsive indicator of quality care.

## Background

National and international guidelines provide evidence-based advice about the management of acute asthma, emphasising the need for objective assessment, prompt treatment of the attack, and provision of self-management education as part of structured follow-up. [1,2] Despite early hopes about the potential of guidelines to improve practice,[3] and increasing emphasis on ensuring wide dissemination,[4,5] there is continuing concern that the care of acute asthma remains suboptimal. [6-9] This is of particular importance in primary care where 90% of acute asthma is managed. [6-8]

Acute asthma is common,[10] with over 100,000 admissions and 1,500 deaths a year attributed to asthma in the UK. [11,12] Confidential enquiries into the cause of asthma deaths over the last three decades have consistently implicated failure to appreciate the severity of the attack resulting in delayed, inadequate emergency treatment as a potentially preventable factor. [13-16]

Didactic lectures and written guidelines, even those including a brief summary of relevant points for clinicians, are known to be ineffective in promoting change in practice [17]. This has stimulated the development of less passive educational interventions. Audit and feedback can change professional practice, though the magnitude of effect varies and questions remain about the most appropriate supporting interventions[18]. Recent policy-driven changes in continuing medical education have led to a shift towards formal needs assessment and multi-professional practice-based learning in primary care through professional development plans[19]. Founded on these concepts a Professional Development Programme was developed by the General Practice Airways Group (a UK primary care interest group). The programme provides a framework for practices wishing to improve their management of acute asthma which incorporates audit, feedback and an interactive workshop. An early pilot study using the programme in three selected, asthma-interested practices, suggested it may have the potential for positively changing practice behaviour[20].

Our randomised controlled trial aimed to establish the effectiveness of the Acute Asthma Professional Development Programme to improve management (specifically the recording of objective assessment of severity) in general practices recruited from one region of Scotland.

## Methods

The trial was undertaken during 2002 with approval from Grampian Research Ethics Committee.

### Recruitment of practices

We invited, by letter, all 59 general practices in the Aberdeen, and Banff and Buchan regions of Grampian, Scotland to participate in the study. All non-responding practices were telephoned and a personal invitation issued. Participating practices gave their fully informed consent to taking part in all aspects of the development programme.

### Randomisation

Using random number tables, consenting practices were centrally randomised to early or delayed intervention groups by a researcher not involved in the trial. It was not possible to blind the practices as the intervention was an active process. The researchers were aware of allocation, though the audit data were submitted to a different centre (Tayside Centre for General Practice, Dundee) to that of the research team organising the educational intervention in Aberdeen.

### Procedure

Each enrolled practice undertook audits of their management of acute asthma at baseline, 6-months and 12-months using previously piloted methodology[20]. We provided full instructions on how to undertake the audits and a study helpline was available for support. The educational intervention included feedback of audit data and attendance at a workshop during which a practice development plan was formulated by participants. The intervention was provided immediately after the baseline audit to the early intervention group, and immediately after the 6-month audit to the delayed group.

### The Acute Asthma Professional Development Programme

#### The baseline audit

**Figure 1:**
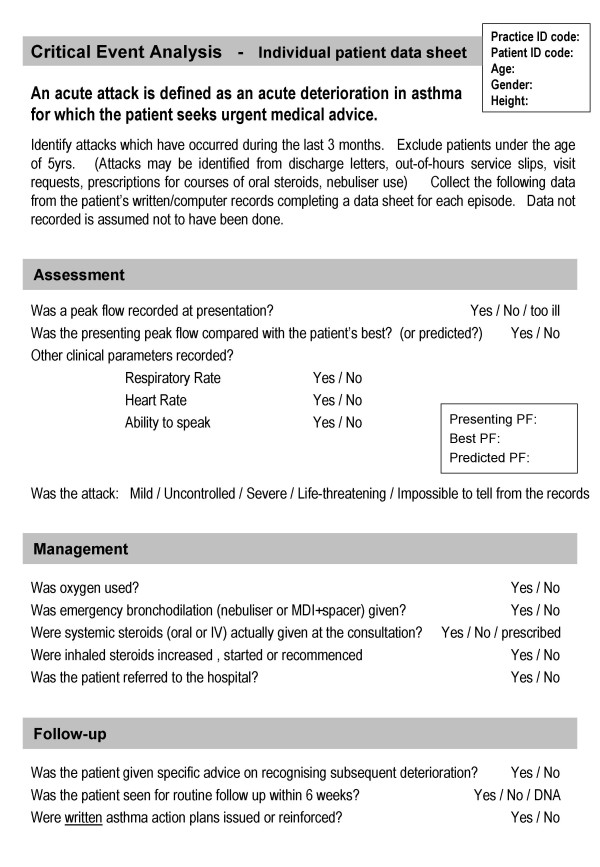
Audit form for the critical event analysis.

Practices undertook a critical event analysis of acute attacks occurring over a 3-month period in adults and children aged 5 years and over. An attack was defined as "an acute deterioration of asthma for which the patient seeks urgent medical advice"[8]. Participants were advised to identify prospectively all acute attacks occurring during the audit period using computer databases, discharge letters, out-of-hours service slips, visit requests, prescriptions for courses of oral steroids and nebuliser use. Data about the management of the attacks was collected retrospectively by a member of the practice team from patients' written and computer records. The previously piloted critical event analysis form (figure 1) was designed to collect data on objective assessment of severity, treatment provided by health professionals and follow-up within 6 weeks post exacerbation[20]. Actions not recorded were assumed not to have been done.

#### The educational intervention

**Figure 2:**
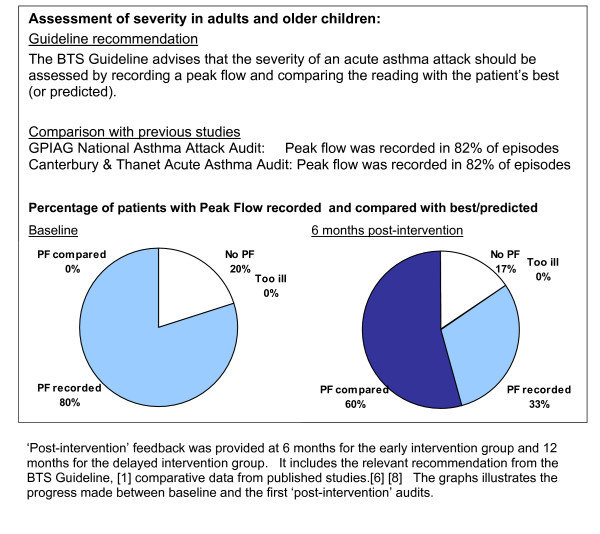
Example of 'post-intervention' feedback provided to practices.

**Figure 3:**
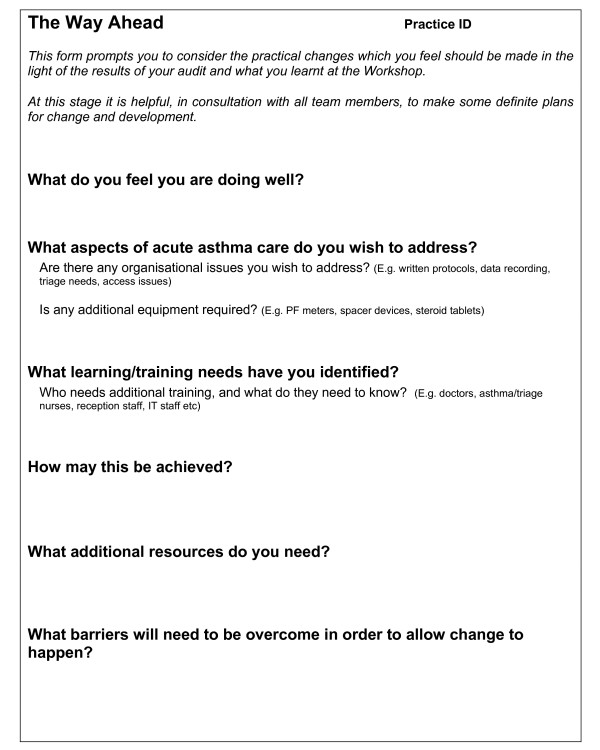
Proforma to facilitate the formulation of a Practice Development Plan.

• Feedback of audit results: The results of the baseline audit were fed back to participating practices with anonymised comparative data from the other practices taking part in the programme, as well as the standards set by current asthma guidelines. Figure 2 provides an example of the feedback which was posted to practices prior to the workshop.

• Multi-disciplinary interactive workshop: A 3-hour workshop held in the University of Aberdeen, facilitated by two of the researchers (a general practitioner (HP) and respiratory nurse (GH)), was attended by representatives of the participating practice teams (normally a GP and practice nurse; the practice manager or senior receptionist was also invited). Amalgamated audit results from the group were discussed and used to tailor discussion towards specific aspects of acute asthma care identified as falling short of guideline standards. Case studies were used to facilitate discussion of practical aspects of acute asthma management highlighting key deficiencies reported in published literature[8]. Recording of objective assessment of attacks was emphasised.

• A list of suggested references (selected because of their relevance to primary care management of acute asthma), resources (e.g. guideline summary charts, web-sites of professional organisations, training organisations, equipment manufacturers) and practical materials (e.g. examples of asthma action plans) were provided to all participants and discussed in the workshops.

• Formulation of a development plan: Time was allocated at the end of the workshop for practice teams to formulate a practice-specific acute asthma development plan. Using an outline proforma, (Figure 3) the practice teams reflected on their current performance, identified aspects of care they wished to improve and made practical plans to overcome barriers and institute change.

#### Follow-up audits

The audits completed at 6-months and 12-months were undertaken using the same methodology as at baseline and were fed back to all practices. The 6-month audit provided a review of progress for the early intervention group, and baseline assessment for the delayed intervention group. The time scale of 6 months had proved to be feasible in our pilot study[20].

### Outcome measures

Our primary outcome measure was the proportion of acute episodes with a recording of a peak flow compared to the patients' best (or predicted if best was not known) at the 6-month audit. This measurement is recommended by current asthma guidelines as a basis for the classification of severity of acute attacks and for determining appropriate emergency treatment[1]. Our pilot study demonstrated an improvement in the proportion of patients with a peak flow compared to best/predicted from the baseline prevalence of 21% to 61% at 6 months[20].

Other outcome measures, reflecting the recommendations of the guideline in force at the beginning of the trial,[21] from the critical event analysis were considered in three domains: assessment (recording of peak flow, 'peak flow compared to best/predicted', respiratory rate, heart rate, ability to speak), management (provision of oxygen, bronchodilation. systemic steroids actually administered, steroids prescribed, inhaled steroids, referral to hospital) and follow-up (provision of advice, follow up consultation, self-management education). Combined scores for each domain were calculated by summing the questions which were answered 'yes' to give a score out of 5, 6 or 3 for assessment, management and follow-up respectively.

### Sample size calculation and statistical analysis

In order to take account of the clustering by practice, the sample size calculation needed to include an estimate of the intracluster correlation coefficient (ICC). Our pilot work suggested that we could expect three acute episodes per full time general practitioner during each three month period and we estimate an average of 5 general practitioners per practice. Using a conservative estimate for the ICC of 0.05, to detect a change of 30% in the proportion with a peak flow compared to best/predicted from a baseline prevalence of 21% with 80% power and 5% two-sided significance level would require 5 practices per arm (ie. an estimated 25 general practitioners and 75 clinical episodes in each group). To allow for withdrawals we decided to recruit at least 20 practices in total.

We analysed associations between categorical data using the chi-square test. Differences in normally distributed combined scores between early and delayed intervention groups were examined with independent sample t-tests.

Because randomisation was at practice level, the statistical analysis had to adjust for the effect of clustering. Multilevel modelling was inappropriate since the outcomes were practice level data across time and not multiple measurements across time on the same individuals. Therefore, the primary analysis was based on the practice summary measures. Analysis of covariance was used to examine differences in outcomes between groups at 6-months and 12-months after adjustment for baseline differences and practice effects. Practices who recorded no exacerbations during an audit cycle were excluded from the analysis at that time point.

## Results

Of the 59 practices invited, 23 (39%) were recruited into the study: those who declined did so due to pressures of time and resources. Practice demography was similar for the two groups at baseline.(Table 1).

**Table 1:** Practice demography

	**Early (n = 11)**	**Delayed (n = 12)**	**p-value**
Median (interquartile range)			

Number of partners	5 (3.0, 7.0)	5 (4.0, 8.8)	0.80
Number of practice nurses	3 (2.0, 4.0)	3 (2.0, 4.0)	0.56
Number of nurses with asthma training	1 (1.0, 1.0)	1 (0.3, 2.0)	0.79

Inner city	2	3	
Urban	3	3	
Semi rural	3	3	
Rural/remote	3	3	

### Withdrawals

**Figure 4:**
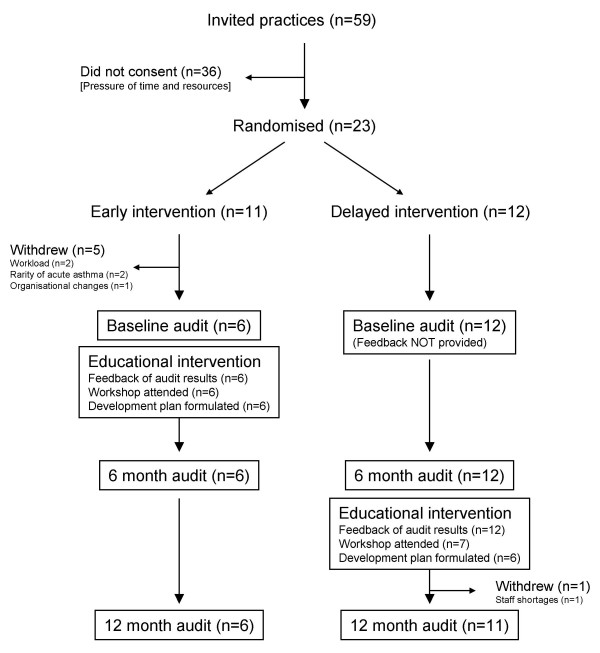
Flow of participating practices through each stage of the trial.

The flow of practices through the trial is given in Figure 4. Five practices in the early intervention group withdrew before submission of the baseline audit data: the remaining six practices completed all aspects of the programme. All but one of the 12 practices in the delayed group submitted data at all three stages (baseline, 6-months and 12-months), but only seven attended the workshop. Practice workload pressure (2), staffing problems (2) or a very low asthma attack rate (2) were the reasons given for withdrawal.

### Critical event analysis

Data were returned from audits at baseline, 6-months and 12-months by the early intervention group on 54, 62, and 86 acute episodes, and by the delayed intervention group on 133, 112, and 98 acute episodes (there were less episodes in the early group because of the greater number of practice withdrawals) GPs in the early intervention group identified a mean of 2.1 episodes in each of the 3-month audit periods compared to 2.0 per GP in the late intervention group There were no significant differences in the age and gender of the patients in the three audit phases, nor between groups (Table 2).

**Table 2:** Comparison of audit data at baseline, 6 m and 12 m in 'early' vs. 'delayed' groups

	**Baseline audit**			**6-month audit**			**12-month audit**		
	**Early**	**Delayed**	**p-value**	**Early**	**Delayed**	**p-value**	**Early**	**Delayed**	**p-value**

Number of episodes submitted	54	133		62	112		86	98	
Mean age (SE)	38.41 (3.66)	39.82 (1.91)	0.71	33.15 (2.73)	36.04 (1.87)	0.37	38.28 (2.68)	36.44 (1.89)	0.98
Gender: male % (n)	44 (24)	32 (42)	0.12	53 (33)	46 (52)	0.39	43 (37)	32 (31)	0.11

Peak flow compared with best									
Unadjusted data % (n)	28 (15)	33 (44)	0.48	42 (26)	29 (32)	0.07	77 (66)	37 (36)	<0.001
Adjusted data %*	-	-	-	54	34	0.18	79	45	0.07

*Using analysis of covariance, p-value refers to the significance between groups after adjustment for baseline value and practice

### Early vs. delayed intervention groups

#### Primary outcome measure: proportion with peak flow compared to best/predicted

The proportion of episodes in which the presenting peak flow had been compared to best (or predicted) was comparable in the early and delayed intervention groups at baseline (see Table 2). There was a trend to continuing improvement in the recording of peak flow compared to best/predicted in the early group practices throughout the year. Unadjusted data showed no difference at 6-months (p = 0.07) but a statistically significant improvement was observed at 12-months (p < 0.001). Although this trend remained, these differences did not reach significance at either time point after adjustment for baseline and practice effects.

#### Combined scores

The mean combined scores are given in Table 3. In the early intervention group, there was a consistent trend to gradual improvement in assessment and follow-up scores over the year of the study, with highly significant between-group differences in the unadjusted scores at 12-months. After adjustment for baseline difference and practice, the between-group difference in the combined assessment score remained significant at 12-months. There was no consistent change in the management score within or between groups.

**Table 3:** Comparison of combined assessment, management and follow-up scores at baseline, 6-months and 12-months in the early vs. delayed groups

Values are mean (SE)						
	**Unadjusted data**			**Data adjusted for baseline values and practice**		

	**Early**	**Delayed**	**P-value**	**Early**	**Delayed**	**P-value**

**Combined assessment score**						
Baseline	1.94 (0.24)	2.00 (0.13)	0.83	-	-	-
6-months	2.44 (0.18)	1.75 (0.12)	<0.01	2.48 (0.43)	2.26 (0.33)	0.69
12-months	3.37 (0.15)	2.07 (0.15)	<0.001	3.60 (0.35)	2.30 (0.28)	0.02

**Combined management score**						
Baseline	2.07 (0.14)	2.15 (0.08)	0.04	-	-	-
6-months	2.19 (0.16)	2.30 (0.09)	0.03	2.15 (0.21)	2.26 (0.16)	0.67
12-months	1.94 (0.11)	1.92 (0.11)	0.95	1.91 (0.40)	2.44 (0.32)	0.32

**Combined follow-up score**						
Baseline	1.06 (0.12)	0.91 (0.09)	0.36	-	-	-
6-months	1.58 (0.12)	0.94 (0.08)	<0.001	1.71 (0.25)	1.38 (0.19)	0.31
12-months	1.81 (0.09)	1.04 (0.10)	<0.001	1.84 (0.25)	1.91 (0.20)	0.82

**Figure 5:**
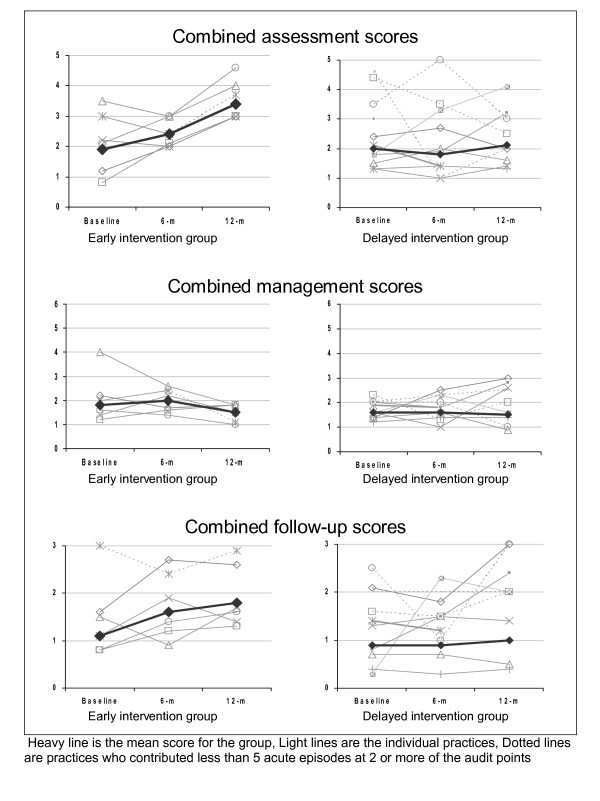
Timescale of changes in combined scores within the early and delayed intervention groups.

The timescale of the changes in the combined assessment, management and follow-up scores in the individual practices are illustrated in Figure 5. The most consistent effect was the improvement in the combined assessment scores in early intervention practices.

### Formulation of practice development plans

Twelve practices (6 'early', 6 'delayed') returned their completed practice development plans. Aspects of care they hoped to improve included the assessment process (5 'early', 1 'delayed'), follow up provision (3 'early', 3 'delayed'), the use of self-management plans (4 'early' 3 'delayed') and the use of oxygen (1 'early' 3 'delayed').

## Discussion

Our practice development programme incorporating audit, feedback and a workshop did not result in a significant improvement in our primary outcome measure (the proportion with peak flow compared to best/predicted) at either the 6 or 12 month time points after adjustment for baseline and practice effects. However, one of our secondary outcome measures (the recording of the objective assessment of attacks) showed a trend to improvement which was apparent at 6-months, and reached statistical significance at 12-months, suggesting that the hypothesised 6-month period was too short to enable practices fully to implement change. The marked trend to improvement in follow up arrangements lost its statistical significance after adjustment for practice effects and baseline differences. The latter, however, may be an over-adjustment as collecting and responding to the baseline audit data was a part of the educational intervention. For example: poor baseline performance should stimulate a practice to develop that aspect of their care. The true effect of this complex intervention is, therefore, likely to fall somewhere between our adjusted and unadjusted results.

### Limitations of the study

The withdrawal of five practices from the early intervention group before submitting their baseline data was unfortunate. The remaining practices are likely to have been the most motivated making it easier to effect change in this group. By contrast, all but one of the delayed intervention group practices completed the programme, though five did not attend the educational intervention. If less motivated practices in the 'delayed' group had chosen to 'stay the course' rather than withdraw this might have diluted any effect in this group. However, because of the considerable commitment required we believe it is unlikely that reluctant practices would have continued to provide data.

We rejected the option of collecting baseline data before randomisation because the audit was an integral part of the educational intervention. Willingness to participate in all aspects of the programme (including the audit, workshop and development plan) was one of the factors potentially influencing the effectiveness of this 'complex intervention'[22] Randomising only those practices who submitted baseline data would have eliminated one of these important factors.

Practices submitted less than the projected three episodes/GP in each three month audit period. This, combined with the withdrawal of five practices, meant that we did not achieve the required number of 75 acute episodes at baseline and 6-months in the early intervention group so we were slightly underpowered to detect a statisticallysignificant change in the primary outcome measure. Although raising the possibility that some acute episodes had been overlooked, the rate of identification of acute attacks per GP was similar in both groups, and was consistent over the three audit time points. In addition, the demography of the patients suffering acute episodes was similar to that described in previous regional and national audits (allowing for the slightly different recruitment strategies). [6-8] The reduction in number of acute episodes may reflect the reported decline in asthma exacerbations in the UK[23]. The audits were carried out internally by practice staff, which introduced the possibility of inaccurate, incomplete or biased data. Despite this concern, we considered it important that practices undertook their own data collection as part of the learning process. Financial constraints prevented quality checks of the accuracy of submitted data.

Of the 59 practices approached, just over a third agreed to participate, and just over a quarter completed the programme which may be interpreted as limiting generalisability. However, the recruited practices reflected the range of demography typical of the area. In addition, participation in a professional development plan is an active process which demands considerable application with significant workload implications for members of the team. The reasons given for non-participation and withdrawal suggest that the time and effort required was not possible for many practices during the study period. Practice development needs vary over time and it is likely that under other circumstances other practices would have wished to participate.

### Main strengths of study

Our trial included a broad range of urban and rural practices, though to facilitate local workshops we limited recruitment to one Scottish region. The demography of the practices in the two study groups was similar. The audit tools were developed from a published survey,[8] and the proforma had been used successfully in an earlier pilot project[20]. The data submitted from the baseline audit was broadly similar to that observed in other published surveys [6-8,20] increasing confidence in the reliability of our outcomes.

### Interpretation of findings in relation to previously published work

In common with the majority of studies of educational interventions, [24,25] our trial evaluates 'behaviour' – the third tier of Kirkpatrick's hierarchy of levels of evaluation[26]. Although this is one step removed from the ultimate goal (improved patient outcomes) our selected process outcomes are recommended by evidence based guidelines[21]. Importantly, poor performance in objectively assessing severity has been consistently linked with poor patient outcomes in a number of confidential enquiries. [13-16] In addition, keeping adequate records which allow retrospective assessment of severity is good clinical practice and a medico-legal requirement. [27,28] The development plans prepared by individual practices confirmed that objective assessment was a priority for five of the early intervention group practices. Referral data, a potential outcome measure which we included within the management domain, is difficult to interpret as rates could be influenced by management strategies or different severity of presenting attacks.

Using the structure of Professional Development Plans [19] our intervention not only involved the recognised formula of 'audit and feedback', [18] but also sought formally to engage the practice team in the process of identifying the challenges and obstacles relevant to their practice, and to plan how change may be effected. This is a recognised educational strategy,[25,29] but 'individualising' of the practice development plan, but may have diluted the effect on specific outcomes as some practices may have decided that certain aspects were not relevant to them. Whilst incorporating many of the key elements of the stepwise cycle of change described by Grol,[30] we may have underestimated the importance of the 6-month audit. Audit is associated with improved asthma care,[31] and any improvements at 6-months (albeit not significant) may have encouraged the 'early' practices to further develop their care in time for the 12-month audit. By contrast the delayed intervention group received no feedback until the 6-months and had little time to effect change.

There is no consensus on the ideal duration of educational intervention studies,[18] though six months is the median duration of trials reviewed by Wensing,[32] Ideal duration is a balance between the time needed to effect change, whilst avoiding the danger of deterioration if the time scale were too prolonged[18]. Our trial was designed to demonstrate a difference between the two groups at 6-months, when the early intervention group had received the education component of the programme with the 'delayed' practices acting as controls. However, after adjustment for baseline differences and practice, statistically significant change only occurred at 12-months. Future randomised trials of educational interventions need to recognise that effecting change in practices takes time. Although the timeframe for change was implicit within the structure of the trial, the addition of an explicit timeline within the Practice Development Plan might have facilitated more rapid change.

In order to achieve improvement, the practices had to commit to the project for a year and participate in three audit cycles and a workshop. We offered no financial incentives to cover the cost of the work involved suggesting that the process of formulating a practice-specific acute asthma development plan in response to their baseline performance had sufficiently engaged practices to encourage an on-going focus on aspects of care they wished to improve.

Other factors which may have influenced outcomes include the publication of national guidelines in February 2002,[1] though this is unlikely to have significantly affected our results as the final audit was already underway at the time of the launch. The primary care management of acute asthma was not one of the key messages promoted by the publicity following the launch[4]. The lack of substantial change in the delayed intervention group confirms that this was unlikely to be a significant factor.

Interpretation of the management scores is difficult without objective independent assessment of asthma severity. It is possible that the more aggressive management of attacks at baseline reflected more severe attacks at this audit time point rather than inappropriate management.

## Conclusion

Although our randomised trial of an acute asthma professional development programme for general practices did not demonstrate significant improvements at the a priori 6-month assessment point, there was an improvement compared to baseline in the objective assessment of severity 12 months into the trial. Monitoring of the assessment of acute attacks proved to be a feasible and responsive indicator of quality care. Our findings, especially the timescale needed to effect change, have implications for the design of future trials.

## Competing interests

The author(s) declare that they have no competing interests.

## Authors' contributions

HP initiated the idea for the study and led the development of the protocol, supervised the study, undertook data analysis, interpretation of results and writing of the paper. JF led the study administration and carried out the study. GH and BS undertook data analysis and feedback. AL supervised the multivariate analyses and helped with interpretation of results. DP provided advice on the development of the protocol, on-going support and interpretation of the results. All authors reviewed and approved the final manuscript. JF and HP are study guarantors.

## Pre-publication history

The pre-publication history for this paper can be accessed here:


